# Metabolomic analysis of rat serum in streptozotocin-induced diabetes and after treatment with oral triethylenetetramine (TETA)

**DOI:** 10.1186/gm334

**Published:** 2012-04-30

**Authors:** Marta Ugarte, Marie Brown, Katherine A Hollywood, Garth J Cooper, Paul N Bishop, Warwick B Dunn

**Affiliations:** 1 School of Biomedicine and Manchester NIHR Biomedical Research Centre, AV Hill Building, The University of Manchester, Oxford Road, Manchester, M13 9PL, UK; 2 Centre for Advanced Discovery and Experimental Therapeutics, School of Biomedicine, University of Manchester and Manchester Academic Health Sciences Centre, Central Manchester NHS Foundation Trust, York Place, Oxford Road, Manchester, M13 9WL, UK; 3 Maurice Wilkins Centre for Molecular Biodiscovery, School of Biological Sciences, Thomas Building, The University of Auckland, Private Bag 92019, Auckland Mail Centre, Auckland 1142, New Zealand; 4 Department of Pharmacology, University of Oxford, Mansfield Road, Oxford, OX1 3QT, UK; 5 Manchester Centre for Integrative Systems Biology and School of Chemistry, Manchester Interdisciplinary Biocentre, University of Manchester, 131 Princess Street, Manchester, M1 7DN, UK

## Abstract

**Background:**

The prevalence, and associated healthcare burden, of diabetes mellitus is increasing worldwide. Mortality and morbidity are associated with diabetic complications in multiple organs and tissues, including the eye, kidney and cardiovascular system, and new therapeutics to treat these complications are required urgently. Triethylenetetramine (TETA) is one such experimental therapeutic that acts to chelate excess copper (II) in diabetic tissues and reduce oxidative stress and cellular damage.

**Methods:**

Here we have performed two independent metabolomic studies of serum to assess the suitability of the streptozotocin (STZ)-induced rat model for studying diabetes and to define metabolite-related changes associated with TETA treatment. Ultraperformance liquid chromatography-mass spectrometry studies of serum from non-diabetic/untreated, non-diabetic/TETA-treated, STZ-induced diabetic/untreated and STZ-induced diabetic/TETA-treated rats were performed followed by univariate and multivariate analysis of data.

**Results:**

Multiple metabolic changes related to STZ-induced diabetes, some of which have been reported previously in other animal and human studies, were observed, including changes in amino acid, fatty acid, glycerophospholipid and bile acid metabolism. Correlation analysis suggested that treatment with TETA led to a reversal of diabetes-associated changes in bile acid, fatty acid, steroid, sphingolipid and glycerophospholipid metabolism and proteolysis.

**Conclusions:**

Metabolomic studies have shown that the STZ-induced rat model of diabetes is an appropriate model system to undertake research into diabetes and potential therapies as several metabolic changes observed in humans and other animal models were also observed in this study. Metabolomics has also identified several biological processes and metabolic pathways implicated in diabetic complications and reversed following treatment with the experimental therapeutic TETA.

## Background

Diabetes mellitus (DM) is a chronic debilitating condition that is rapidly increasing in prevalence worldwide, as a consequence of increases in obesity, changing patterns of diet and physical activity, and ageing populations. The World Health Organization estimated that 154 million people in the world had DM at the beginning of the 21st century [1]. In the USA the prevalence is estimated to increase from 4.0 to 7.2% (or 29 million) between 2000 and 2050 [2].

DM is a metabolic disorder characterized by hyperglycemia. The hyperglycemia is caused as a consequence of a deficiency in insulin in type 1 diabetes (T1D), and is a feature of late type 2 diabetes (T2D) along with insulin resistance. T2D is significantly more prevalent than T1D. Molecular pathophysiological mechanisms that precede hyperglycemia, or are observed with the clinical symptoms of DM, include, among others, alterations in lipid and amino acid metabolism [3-5], changes in hormone levels (including insulin [6] and adiponectin [7]), increases in adipokine levels [8] and alterations in copper metabolism [9]. The complications of DM include cardiomyopathy, vasculopathy, neuropathy, nephropathy and retinopathy, and are major causes of morbidity and mortality. Current interventions in DM are aimed at controlling blood glucose levels, dyslipidemia and blood pressure, but these have only modest effects on reducing risk of progression to complications, so better treatments are urgently required.

DM is a disease associated with multiple metabolic abnormalities so the application of metabolomic techniques to study it and potential interventions is appropriate [10]. A recent review has highlighted the role of metabolomics in the study of DM and cardiovascular diseases [11]. Previous studies have reported alterations in biofluid or tissue metabolic profiles in humans [3-5] and experimental animal models of DM (obese Zucker rat [12], db/db mouse [13], ddY-H mouse and streptozotocin (STZ) rat [14,15]) applying metabolomics.

A number of animal models have been used to study DM [16] and here we chose to apply the commonly used STZ rat model. STZ is a toxin with the ability to induce selective destruction of pancreatic beta cells resulting in insulin deficiency and hyperglycemia [17]. In the STZ rat model, the concentration of insulin decreases rapidly after STZ-induced beta cell destruction and the blood glucose levels increase to greater than 11 mmol/L. The STZ rat model of diabetes has been widely investigated since 1963 and is one of the most commonly used models of human disease. It is known to mimic many of the acute and some of the chronic complications observed in human diabetes. This model has the advantage of being highly reproducible and the time lines for various complications to develop are well recognized and reproducible. Given the established similarities of some of the structural, functional and biochemical abnormalities to human disease, it is considered an appropriate model to assess mechanisms of diabetes and evaluate potential therapies.

One potential treatment under investigation to treat diabetic complications (though not to reverse hyperglycemia) is the copper (II)-selective chelator triethylenetatramine (TETA) [18]. Recent research has described elevated plasma and urine concentrations of copper in human and experimental DM (including the STZ-diabetic rat model [19]) and copper concentrations are highest in subjects with diabetic complications such as retinopathy and nephropathy. Retention of copper has been shown in the kidney [19], liver [19] and heart [20] in DM and plays a role in increased cellular oxidative stress through enhanced production of reactive oxygen species (in particular the hydroxyl radical) through Haber-Weiss Fenton reactions. Oral treatment with copper chelators has been shown to reverse DM-induced changes and restore copper homeostasis (reviewed in [18]). The copper (II)-selective chelator TETA, which is used as a second line treatment for Wilson's disease, ameliorates cardiomyopathy [20,21] and diabetes-induced nephropathy [22]. Furthermore, a recently completed phase 2a clinical trial has shown TETA to be well tolerated in DM and to improve hyperglycemia-induced left ventricular hypertrophy and diastolic dysfunction [21]. TETA has also been demonstrated to have anti-angiogenic properties and its potential use in cancer chemotherapy is under investigation [23].

The two primary objectives of the research described were to (i) assess the metabolic changes in the STZ-induced rat model of DM and compare these changes to metabolic changes observed in published research in relation to other animal models of DM and (ii) to investigate the metabolic response to TETA treatment in the STZ-induced rat model of DM. Serum was chosen as an appropriate biofluid to integrate the diabetes-induced changes that occur in multiple tissues. The investigation of the serum metabolome was chosen as DM is defined as a metabolic disorder and changes in metabolism are expected. The application of metabolomics to study the effect of TETA treatment on the reversal of diabetic complications is also appropriate; the mode of action of TETA is to chelate copper and reduce oxidative stress in cells and tissues. Markers of oxidative stress (including oxidized lipids) are expected to be observed in the serum metabolome. This study had the potential to identify metabolic biomarkers to apply in other studies - for example, the monitoring of drug safety and efficacy in clinical trials. Two independent but similar biological studies were performed at different times, each assessing metabolic differences in four groups: (i) non-diabetic/untreated rats, (ii) non-diabetic/TETA-treated rats, (iii) STZ-induced diabetic/untreated rats and (iv) STZ-induced diabetic/TETA-treated rats. Ultraperformance liquid chromatography-mass spectrometry (UPLC-MS) and univariate/multivariate data analysis methods were applied to determine differences in the serum metabolomes associated with STZ-induced DM and TETA treatment.

## Results

Treatment of the rats with intraperitoneal STZ resulted in elevated plasma glucose levels consistent with diabetes (>30 mmol/L), whereas non-treated rats without STZ injection had normal glucose levels (<7 mmol/L) in each of the two studies. Time-independent replicate studies were performed with metabolomic analyses undertaken in November 2009 (study 1) and May 2010 (study 2). Half the rats in the non-diabetic and diabetic groups were treated with TETA, that is, there were four groups in total. In studies 1 and 2 the numbers of rats, which were present in each of the four classes at time of sacrifice, were as follows: non-diabetic/untreated (study 1, n = 9; study 2, n = 10), nondiabetic/TETA-treated (study 1, n = 6; study 2, n = 10), STZ-induced diabetic/untreated (study 1, n = 9; study 2, n = 10) and STZ-induced diabetic/TETA-treated (study, 1 n = 6; study 2, n = 10). Following quality assurance procedures, 4,826 and 7,425 metabolic features were detected in positive ion mode in studies 1 and 2, respectively, and 1,369 and 3,469 metabolic features in negative ion mode in studies 1 and 2, respectively. These metabolic features were taken forward for further data analysis. Of these metabolic features, 51.9% and 40.3% of metabolic features were putatively identified in the two studies, respectively, following the process of metabolite identification. This highlights that not all metabolites are identified in untargeted metabolic studies.

**Figure 1:**
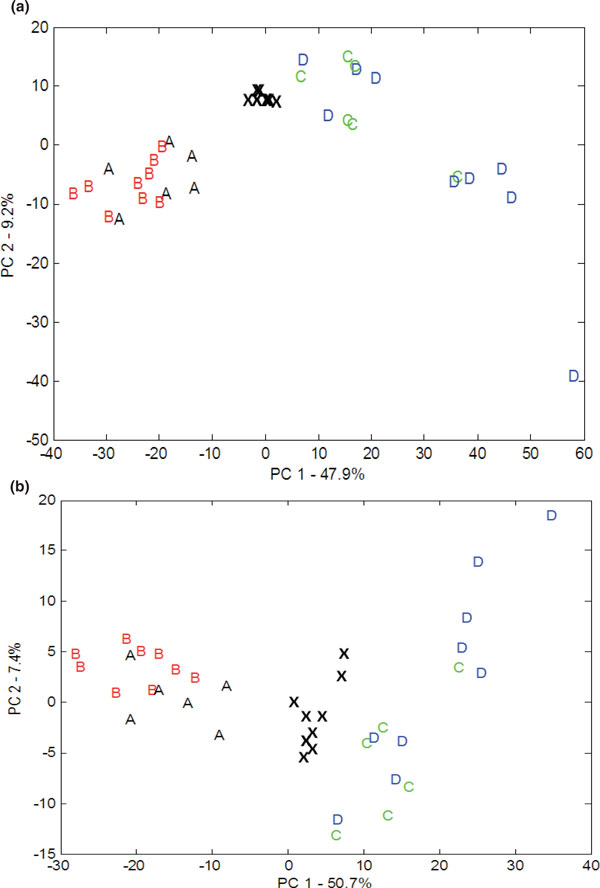
**Principal components analysis (PCA) score plots**. **(a,b) **Data acquired in positive (a) and negative (b) ion modes in independent study 1. The letter 'A' represents non-diabetic/triethylenetetramine-treated, 'B' non-diabetic/untreated, 'C' STZ-induced diabetic/triethylenetetramine-treated, 'D' STZ-induced diabetic/TETA-untreated and 'X' the QC samples.

Principal components analysis (PCA) was performed applying all detected metabolic features to assess the variability in the data and determine whether outliers were present. Figure 1a,b shows the PCA score plots (PC1 versus PC2) for positive (Figure 1a) and negative (Figure 1b) ion mode data acquired in study 1. One potential outlier was identified in positive ion mode only, most probably a result of a poor injection as the total peak area for all metabolites was lower than for other samples, and was removed prior to univariate data analysis. The quality control (QC) samples are tightly clustered in comparison to the rat serum samples, showing that the reproducibility of data acquired in a single UPLC-MS experiment is high. Distinct and clear separation in PC1 indicates a large difference in the serum metabolome related to the differences between STZ-induced diabetic rats and non-diabetic rats, independent of whether rats were treated or untreated with TETA. No clear separation was observed when comparing STZ-induced diabetic rats that were TETA treated or untreated and no clear separation was observed when comparing non-diabetic rats that were TETA treated or untreated. Similar observations were observed in study 2.

Univariate statistical analyses were performed to identify metabolic features whose relative concentrations were statistically different (*P *< 0.05) between different pairwise combinations of the four study groups. A single metabolite can be detected as different metabolic features, each with the same retention time but a different *m/z *value caused by the detection of different ionic species of the same metabolite (for example, protonated and sodiated ions). Also, a single metabolic feature can relate to multiple stereoisomers. Therefore, multiple metabolic features can correspond to a single metabolite or stereoisomer. The number of statistically significant (*P *< 0.05) metabolic features for each comparison and animal study and the number of metabolic features observed as statistically significant in both studies are shown in Table 1. When comparing the STZ-induced diabetic/untreated and non-diabetic/untreated rats, a large number of differences in the serum metabolome were observed. A small number of changes were observed when comparing STZ-induced diabetic/TETA-treated against STZ-induced diabetic/untreated rats and non-diabetic/TETA-treated against non-diabetic/untreated rats. A range of metabolic features were observed to be statistically different in only one of the two studies and a subset of metabolic features was reproducibly observed in both independent studies; 49.0 to 97.9% of statistically significant features defined in one study were not reported as statistically significant in the other study and can therefore be defined as false positives. This highlights the potential for false observations in these types of discovery investigations and an appropriate manner in which to reduce the probability of false positives through two separate discovery studies. In summary, as shown for the PCA analysis, the major changes as defined following univariate analysis are related to diabetic status and fewer changes were observed when comparing TETA-treated and untreated rats.

**Table 1:** Summary of the number of metabolic features that show statistically significant differences (*P *< 0.05) in each group comparison for each independent study and the number of features that are statistically significant in both independent studies

Comparison	Study 1 (*P *< 0.05)	Study 2 (*P *< 0.05)	Both studies (*P *< 0.05)
Non-diabetic/TETA-treated rats versus non-diabetic/untreated rats	234	211	5
Non-diabetic/TETA-treated rats versus STZ-induced diabetic/TETA-treated rats	1,129	3,077	576
Non-diabetic/TETA-treated rats versus STZ-induced diabetic/untreated rats	1,310	2,943	623
Non-diabetic/untreated rats versus STZ-induced diabetic/TETA-treated rats	1,480	3,308	768
Non-diabetic/untreated rats versus STZ-induced diabetic/untreated rats	1,677	2,982	791
STZ-induced diabetic/TETA-treated rats versus STZ-induced diabetic/untreated rats	145	406	12

STZ, streptozotocin; TETA, triethylenetetramine.

Table 2 describes the 148 metabolic features whose concentration changes were statistically significant (*P *< 0.05) (i) when comparing STZ-induced diabetic/untreated versus non-diabetic/untreated rats, (ii) in both independent studies and (iii) with the same direction of relative change (up-regulation or down-regulation) in both independent studies. This provides further confidence that the metabolites defined as 'biologically interesting' are relevant and deserve further investigation. Specific metabolite classes that are over-represented in the results include amino acids and related metabolites (10 metabolites), bile acids (5 metabolites), dipeptides (2 metabolites), long- and short chain fatty acids and related metabolites (23 metabolites), glycerophospholipids (41 metabolites), nucleosides, nucleotides and purine metabolites (6 metabolites), sphingolipids (4 metabolites) and vitamin D metabolites (3 metabolites).

**Table 2:** Metabolites shown as statistically significant (*P *< 0.05) when comparing non-diabetic/untreated and streptozotocin-induced diabetic/untreated rats in both independent studies

	Discovery		Validation	
	
Metabolite	*P*-value	Ratio (diabetic/non-diabetic)	*P*-value	Ratio (diabetic/non-diabetic)
**Amino acids and related metabolites**				
Isovalerylalanine AND/OR isovalerylsarcosine	0.00049	8.20	0.00016	2.85
5-Methoxytryptophan	0.00113	7.74	0.00038	2.13
N-[O-Phosphono-pyridoxyl]-isoleucine	0.00150	2.46	0.00115	3.30
Isoleucine AND/OR leucine AND/OR norleucine AND/OR N-methylvaline	0.00035	2.39	0.00067	1.51
Arginine	0.02434	0.67	0.00016	0.54
Beta-cyclohexyl-alanine	0.00411	0.66	0.00320	0.81
Proline	0.00708	0.65	0.00320	0.77
2-Methylserine AND/OR homoserine AND/OR allothreonine	0.00932	0.51	0.00024	0.41
Oxoproline	0.03347	0.45	0.00038	0.50
Tryptophan	0.00035	0.42	0.00016	0.44
				
**Bile acids**				
24-Nor-5beta-cholane-3alpha,7alpha,12alpha,22,23-pentol	0.00127	2.84	0.00016	2.10
24-Nor-5beta-chol-22-ene-3alpha,7alpha,12alpha-triol AND/OR (22R)-3alpha,7alpha,22-Trihydroxy-5beta-cholan-24-oic acid	0.02092	2.14	0.04937	1.71
(22E)-3alpha,6beta,7beta-trihydroxy-5beta-chol-22-en-24-oic acid AND/OR 3,7-dihydroxy-12-oxocholanoic acid	0.03877	1.94	0.03376	3.09
(22R)-3alpha,7alpha,22-trihydroxy-5beta-cholan-24-oic acid	0.03051	1.66	0.02334	1.92
(22E)-12alpha-hydroxy-3-oxochola-1,4,22-trien-24-oic acid AND/OR 12alpha-hydroxy-3-oxochola-1,4,6-trien-24-oic acid	0.00067	0.85	0.00150	0.88
				
**Dipeptides**				
Gamma-glutamyl-L-isoleucine AND/OR gamma-glutamyl-L-leucine	0.00035	3.79	0.00016	1.79
(5-L-Glutamyl)-L-glutamine	0.00035	0.36	0.00016	0.58
				
**Fatty acids and related metabolites**				
Methyl-hexadecanedioic acid AND/OR heptadecanedioic acid AND/OR 9,10-dihydroxy-13-hydroperoxy-11-octadecenoic acid	0.00172	3.41	0.00088	2.05
Dodecenedioic acid AND/OR 9,12-dioxo-dodecanoic acid	0.00093	2.26	0.00067	1.50
Octadecatrienoic acid	0.00708	1.90	0.00250	2.50
Hydroperoxyoctadecadienoic acid	0.02434	1.46	0.01261	1.96
Heptadecenoic acid	0.04694	1.26	0.03429	1.69
Hexadecenoic acid AND/OR methyl-pentadecenoic acid AND/OR dimethyl-tetradecenoic acid	0.02434	0.63	0.00407	0.22
Hydroxydecadienedienoic acid	0.04600	0.56	0.04125	0.75
Hydroxydecanoic acid	0.00150	0.49	0.00051	0.46
				
**Lysophospholipids**				
LysoPE(18:0)	0.03051	1.38	0.04937	1.20
LysoPE(18:2)	0.00708	1.33	0.00016	2.44
LysoPC(18:2)	0.03798	1.17	0.00029	1.26
LysoPC(18:1)	0.01185	0.70	0.02014	0.67
LysoPC(20:4)	0.01255	0.70	0.00021	0.60
LysoPC(16:1)	0.02434	0.65	0.00051	0.21
LysoPC(14:0)	0.00093	0.57	0.00250	0.29
LysoPC(20:1)	0.00541	0.48	0.01261	0.61
LysoPC(22:6)	0.00172	0.46	0.00320	0.46
LysoPC(dm16:0)	0.00035	0.43	0.00088	0.56
LysoPC(O-16:2)	0.02092	0.32	0.01255	0.22
LysoPC(22:5)	0.00146	0.30	0.00021	0.27
				
**Nucleosides, purine and pyrimidine metabolites**				
1,7-Dimethylxanthine AND/OR theobromine AND/OR theophylline	0.00035	3.40	0.00016	2.87
Thionicotinamide-adenine-dinucleotide	0.00035	2.68	0.00021	1.64
5-Methyl-2'-deoxycytidine	0.01255	2.54	0.00016	2.10
Hydroxyadenine AND/OR guanine	0.00049	0.81	0.00021	0.77
Cytosine	0.00049	0.73	0.00021	0.59
Deoxycytidine	0.03389	0.48	0.00016	0.32
				
**Phospholipids**				
PC(36:2)	0.00035	2.95	0.00016	1.76
PE-NMe2(32:0) AND/OR PC(34:1)	0.00049	2.45	0.00115	1.23
PE(36:2)	0.00172	2.28	0.00016	2.47
PE(38:3)	0.00035	2.05	0.00051	1.44
PC(2:0/O-18:0) AND/OR PC(O-16:0/4:0)	0.00310	1.95	0.00250	1.42
PC(34:2)	0.00172	1.91	0.00016	1.38
PC(36:2)	0.00172	1.88	0.00016	1.51
PC(20:2/dm18:1) AND/OR PC(20:3/dm18:0) AND/OR PC(O-16:0/22:4) AND/OR PC(O-18:0/20:4)	0.04550	1.72	0.00082	1.37
PE(38:2)	0.00067	1.59	0.00016	2.26
PC(34:3)	0.00232	1.54	0.00024	2.30
PC(36:4)	0.01185	1.52	0.00194	1.18
PC(6:0/O-16:0) AND/OR PC(O-12:0/10:0) AND/OR PC(O-18:0/4:0) AND/OR PC(O-20:0/2:0)	0.00328	1.52	0.00220	1.62
PC(40:5)	0.00172	1.49	0.00194	1.13
PC(38:5)	0.00932	1.42	0.02837	1.06
PC(40:8)	0.03389	1.26	0.00465	1.31
PC(18:2/dm18:1) AND/OR PC(18:3/dm18:0) AND/OR PC(20:3/dm16:0) AND/OR PC(O-16:0/20:4)	0.04236	1.19	0.01789	1.15
PC(32:1)	0.04694	0.84	0.00016	0.17
PC(36:0)	0.00708	0.70	0.02837	0.90
PC(16:0/O-16:0) AND/OR PC(O-14:0/18:0)	0.02689	0.69	0.00021	0.60
PC(O-8:0/O-8:0)	0.00093	0.62	0.00150	0.63
PC(44:9)	0.03347	0.61	0.00024	0.60
PC(42:7)	0.03389	0.55	0.00016	0.71
PC(18:1/dm18:1) AND/OR PC(18:2/dm18:0) AND/OR PC(20:2/dm16:0) AND/OR PC(O-16:0/20:3) AND/OR PC(20:4/dm18:1)	0.00415	0.53	0.00194	0.63
PC(18:0/O-16:0) AND/OR PC(O-18:0/16:0) AND/OR PC(38:4)	0.01928	0.53	0.04937	0.86
PC(34:4)	0.00218	0.47	0.00024	0.31
PC(38:2)	0.00172	0.43	0.00051	0.60
PC(18:0/dm18:1) AND/OR PC(18:1/dm18:0) AND/OR PC(18:2/O-18:0) AND/OR PC(20:1/dm16:0) AND/OR PC(P-18:0/18:1) AND/OR PC(20:3/dm18:1)	0.00232	0.32	0.00407	0.76
				
**Short chain fatty acids and organic acids**				
Azelaic acid	0.00232	5.22	0.00051	1.68
Oxopentanoic acid AND/OR methyl-oxobutanoic acid	0.00035	4.95	0.00088	1.78
Hydroxybutanoic acid AND/OR methyl-hydroxybutanoic acid	0.01255	4.64	0.00016	3.13
2-Ethylhydracrylic acid AND/OR hydroxy-methylbutyric acid	0.00035	4.18	0.00016	2.42
Methylacetoacetic acid	0.00035	4.14	0.00150	1.40
3-Butenoic acid	0.00035	3.79	0.00016	2.66
2-Methylglutaconic acid AND/OR 2,3-dimethylmaleic acid AND/OR 2-methyleneglutaric acid AND/OR 3-hexenedioic acid	0.00035	3.61	0.00194	1.49
Hydroxyacetone AND/OR propanoic acid AND/OR dihydroxybutyric acid AND/OR 4-deoxyerythronic acid	0.00086	2.66	0.00016	1.51
Hydroxyhexanoic acid AND/OR 2-ethyl-2-hydroxybutyric acid	0.00093	2.51	0.00016	2.13
Glyceric acid	0.01417	2.01	0.00024	3.33
Isonicotinic acid AND/OR nicotinic acid AND/OR picolinic acid	0.01185	0.69	0.00650	0.86
Hydroxybenzoic acid AND/OR salicylic acid	0.02689	0.57	0.04125	0.74
Indolepyruvic acid	0.00150	0.56	0.00061	0.55
Methylindolepyruvic acid	0.03347	0.49	0.00220	0.51
Lactic acid	0.00415	0.34	0.00016	0.56
				
**Sphingomyelines**				
SM(d18:1/18:1)	0.00541	1.73	0.00138	1.42
SM(d18:1/22:1)	0.00919	0.75	0.00815	0.67
SM(d18:1/14:0)	0.01517	0.60	0.00128	0.28
SM(d18:1/22:0)	0.00049	0.53	0.00021	0.47
				
**Vitamin D metabolites**				
1Alpha,25-dihydroxy-19-nor-22-oxavitamin D3 AND/OR 26,27-dinor-3alpha,6alpha,12alpha-trihydroxy-5beta-cholestan-24-one AND/OR linoleyl carnitine	0.00035	2.65	0.00038	3.36
11Alpha-hemiglutaryloxy-1,25-dihydroxyvitamin D3	0.02086	1.39	0.03429	1.30
(22E)-(24R)-1Alpha,24,25-trihydroxy-22,23-didehydrovitamin D3	0.01517	0.44	0.00021	0.29
				
**Other metabolites**				
2,5,7,8-Tetramethyl-2-(2'-carboxyethyl)-6-hydroxychroman -glucuronide	0.03671	6.60	0.01123	5.60
C12 SUGAR	0.00035	5.59	0.00016	5.52
Harman	0.00035	5.00	0.00016	3.77
1-O-Coumaroyl-beta-D-glucose AND/OR 4-O-beta-D-glucosyl-4-hydroxycinnamate AND/OR cis-beta-D-glucosyl-2-hydroxycinnamate AND/OR coumarinic acid-beta-D-glucoside AND/OR trans-beta-D-glucosyl-2-hydroxycinnamic acid	0.00035	3.97	0.00029	3.36
Pteridine	0.00053	3.75	0.00051	2.28
1-(4'-Hydroxyphenyl)ethanol AND/OR 3-methoxybenzyl alcohol AND/OR 4-hydroxyphenylethanol	0.00124	3.59	0.00016	1.86
C6 SUGAR	0.00035	3.36	0.00016	3.35
N-Formylpiperidine AND/OR 2-methylbutyrylglycine AND/OR 3-dehydrocarnitine	0.00127	3.09	0.00016	1.88
Leucine phosphonic acid AND/OR norleucine phosphonate AND/OR N-trimethyl-2-aminoethylphosphonate	0.00146	2.72	0.00341	1.96
2-Amino-2-methylbutanoate AND/OR amino-pentanoic acid AND/OR 4-methylaminobutyrate AND/OR betaine AND/OR isovaline AND/OR norvaline	0.00035	2.64	0.00029	1.60
Sebacic acid AND/OR 1,8-diaminooctane AND/OR 2-amyl 3-butenoic acid AND/OR nonenoic acid AND/OR 4-hydroxynonenal AND/OR 6-methyl-5-octenoic acid	0.00919	2.51	0.00407	1.63
4-Hydroxyphenylacetylglutamic acid	0.00035	2.50	0.00016	3.35
Riboflavin	0.01742	2.21	0.00629	3.51
Neuromedin B (1-3)	0.00049	2.10	0.00088	1.95
12-Hydroxyjasmonic acid 12-O-beta-D-glucoside	0.04123	2.01	0.00239	5.74
N-(2-hydroxy-2S-methyl-ethyl)-16,16-dimethyl-5Z,8Z,11Z,14Z-docosatetraenoyl amine AND/OR N-(2-methoxy-ethyl)-16,16-dimethyl-5Z,8Z,11Z,14Z-docosatetraenoyl amine	0.00179	1.80	0.00114	1.63
4-Hydroxyphenylacetonitrile AND/OR hydroxyindole AND/OR 2,5,6-trihydroxy-5,6-dihydroquinoline AND/OR 3-succinoylpyridine AND/OR adrenochrome AND/OR hippuric acid	0.00067	1.80	0.00320	1.48
4-Hydroxy-2-quinolinecarboxylic acid AND/OR indole-3-glyoxylic acid AND/OR kynurenic acid	0.00937	1.78	0.01789	1.54
2,6-Dimethoxyphenol AND/OR 4-hydroxy-3-methoxy-benzenemethanol AND/OR hydroxytyrosol	0.00950	1.69	0.03389	2.44
Urea	0.00067	1.68	0.00115	1.44
Dolichyl phosphate AND/OR 1,25-dihydroxy-2-nor-1,2-secovitamin D3 AND/OR 1alpha,25-dihydroxy-19-norvitamin D3	0.04486	1.66	0.04331	1.82
2,5,6-Trihydroxy-5,6-dihydroquinoline AND/OR 3-succinoylpyridine AND/OR adrenochrome AND/OR hippurate	0.03051	1.64	0.00194	1.55
Bacteriohopane-32,33,34,35-tetrol	0.03075	1.59	0.00705	1.92
Acetylcarnitine	0.02789	1.56	0.00038	1.90
Palmitoyl glucuronide	0.02689	1.56	0.00016	1.49
2-Phenylethanol glucuronide	0.01928	1.46	0.00051	1.88
Methylnitrosourea	0.00127	1.45	0.00038	1.98
DG(36:4)	0.02689	1.34	0.00115	2.65
1-(9Z,1Z-octadecadienoyl)-2-(10Z,13Z,16Z,19Z-docosatetraenoyl)-3-O-[hydroxymethyl-N,N,N-trimethyl-beta-alanine]-glycerol	0.00310	1.30	0.00016	1.09
(22alpha)-Hydroxy-cholestanol AND/OR 3alpha,7alpha-dihydroxy-5beta-cholestane AND/OR 5beta-cholestane-3alpha,12alpha-diol	0.03051	1.28	0.01261	1.33
3-Amino-3-(4-hydroxyphenyl)propanoic acid AND/OR 3-hydroxyphenylalanine AND/OR threo-phenylserine AND/OR tyrosine	0.04694	0.84	0.00021	0.66
5-Hydroxy-2-polyprenylphenol AND/OR geranylhydroquinone	0.00919	0.80	0.00937	0.71
Glycinamide AND/OR N-acetyldiamine AND/OR N-methylurea AND/OR N-nitrosodimethylamine	0.00067	0.79	0.00250	0.94
Cervonyl carnitine	0.00250	0.63	0.00115	0.54
Betaine citrate	0.00932	0.61	0.01123	0.61
Beta-cyclohexyl-alanine	0.00708	0.58	0.00250	0.81
3-Ureidoisobutyric acid AND/OR alanylglycine	0.00035	0.53	0.00016	0.45
Propenoate AND/OR alanine AND/OR beta-alanine AND/OR sarcosine	0.04694	0.52	0.00150	0.80
Creatinine	0.00035	0.48	0.00016	0.59
N-(3-Phenyl-2-sulfanylpropanoyl)phenylalanylalanine	0.00067	0.48	0.00016	0.56
Creatine	0.03798	0.45	0.00016	0.48
N1-Methyl-2-pyridone-5-carboxamide	0.00270	0.43	0.00109	0.47
All trans decaprenyl diphosphate	0.03389	0.42	0.00114	0.40
2-Methyl-5-hydroxytryptamine AND/OR 5-methoxytryptamine AND/OR cytisine AND/OR N-methylserotonin	0.01255	0.39	0.00021	0.58
Dehydroisocoproporphyrinogen	0.00035	0.36	0.00220	0.23
5-Amino-5-deoxy-cellobiono-1,5-Lactam;6-(alpha-D-glucosaminyl)-1D-myo-inositol;6-(alpha-D-glucosaminyl)-1D-myo-inositol	0.04331	0.31	0.00815	0.71
7,8,7',8'-Tetradehydroastaxanthin	0.00035	0.23	0.00029	0.57

The metabolites are separated into functional classes and arranged in order of fold change of metabolite in study 1. Where multiple metabolic features were reported for a single metabolite the feature with the *P*-value nearest 1 in study 1 is reported. For multiple metabolite identifications for a single metabolic feature, each unique metabolite is separated by AND/OR. The total carbon number and the number of double bonds are indicated for lysophospholipids, phospholipids and sphingomyelins. DG, diacylglycerol; dm, demethyl; PC, glycerophosphatidylcholine; PE, glycerophosphatidylethanolamine; PE-NMe, monomethylphosphatidylethanolamine; SM, sphingomyelin.

The statistical analysis showed a limited number of metabolic features whose change in concentration was statistically significant when comparing STZ-induced diabetic/TETA-treated versus STZ-induced diabetic/untreated rats. These are shown in Table 3. To further assess changes in the complex interactions of metabolites in metabolic networks, we performed pairwise correlation analysis for three groups in study 2: (i) non-diabetic/untreated, (ii) STZ-induced diabetic/untreated and (iii) STZ-induced diabetic/TETA-treated. This was performed to assess potential complex mechanistic actions of TETA not revealed by univariate analysis. Study 2 was selected as a higher number of rats per group survived to 12 weeks compared to study 1. There were 3.4 million pairwise comparisons. Data were further filtered to detail metabolic features that showed (a) a high positive or negative correlation for non-diabetic/untreated rats and for STZ-induced diabetic/TETA-treated rats (arbitrary chosen as > +0.5 or < -0.5) and (b) a change in the correlation coefficient of >0.5 when comparing non-diabetic/untreated rats with STZ-induced diabetic/untreated rats. Pairwise correlations between different metabolic features of the same metabolite were removed from the dataset and metabolites showing changes in ten or more pairwise correlations with other metabolites were passed forward for biological interpretation. The filtering workflow was chosen to investigate the complex metabolic network in operation and to define metabolites that are highly correlated on a pairwise comparison to other metabolites in non-diabetic/untreated rats and that lose a high correlation in STZ-induced diabetic/untreated rats but in which the high correlation returns in STZ-induced diabetic/TETA-treated rats. These features highlight positive changes produced by TETA treatment in diabetic rats and are shown in Additional file 1. Specific classes of metabolites were over-represented in the results, including bile acids (6 metabolites), fatty acids (19 metabolites), glycerophospholipids (37 metabolites), sterol-based metabolites (7 metabolites), vitamin D metabolites (11 metabolites) and sphingolipids (6 metabolites). Multiply-charged species (338 metabolic features) were also over-represented in the results.

**Table 3:** Metabolite shown as statistically significant (*P *< 0.05) when comparing streptozotocin-induced diabetic/untreated and streptozotocin-induced diabetic/triethylenetetramine-treated rats in both independent studies

	Discovery		Validation	
	
Metabolite	*P*-value	Ratio (diabetic treated/non-diabetic treated)	*P*-value	Ratio (diabetic treated/non-diabetic treated)
Hydroxybutanoic acid AND/OR methyl-hydroxybutanoic acid	0.00898	1.25	0.00898	1.78

For multiple metabolite identifications for a single metabolic feature, each unique metabolite is separated by AND/OR.

## Discussion

Diabetes is a multi-factorial metabolic disease. To study metabolic alterations in an experimental, STZ-induced animal model of DM, we applied UPLC-MS based metabolic profiling. Investigation of serum from animals 12 weeks after induction of the diabetes-like insult with comparison to non-diabetic controls, both in the presence and absence of oral treatment with TETA, were performed. Identification of changes in relative metabolite concentrations revealed changes of specific metabolic pathways or areas of metabolism in response to DM and treatment with TETA.

### Changes in the serum metabolome related to molecular pathophysiological mechanisms of diabetes

In addition to the expected hyperglycemia, changes in the serum concentrations of amino acids and related metabolites, bile acids, dipeptides, short and long-chain fatty acids and related metabolites, glycerophospholipids, nucleosides/nucleotides/purine metabolites, organic acids, sphingolipids and vitamin D metabolites were observed. These metabolic changes could be a result of mechanisms related to DM and associated tissue-specific complications but also unexpected secondary actions of treatment with STZ. However, the findings of our study are compatible with known altered mechanisms in DM, making it reasonable to believe that these changes are related to the diabetic state. A selection of biologically important alterations related to potential tissue-specific changes and observed in human and other animal models of DM will be discussed below. These previously observed changes highlight the applicability of the STZ-induced rat model to the study of metabolic perturbations in DM.

Changes in branched-chain amino acid metabolism related to altered catabolism have been reported previously in the pre-diabetic state in humans [3,5] and in animal models [24]. In our study, increased concentrations of leucine and/or isoleucine as well as isovalerylalanine and/or isovalerylsarcosine (applied as a biomarker of isovaleric acidemia) in the diabetic rats indicate disturbances to branched-chain amino acid metabolism. Connor and colleagues [13] observed changes in branched-chain amino acid and isovaleryl-amino acids in the urine of diabetic db/db mice. Leucine has effects on different processes that can relate to insulin resistance and glucose intolerance and include hepatic gluconeogenesis, pancreatic beta cell function, intracellular mammalian target of rapamycin (mTOR) signaling, and the generation of intermediates that are potentially toxic to mitochondrial function [25]. One potential intervention being investigated for DM is metabolic Roux-en-Y gastric bypass, which surprisingly appears to reverse symptoms and complications in morbidly obese diabetic patients [26]. The current intriguing question as to why gastric bypass surgery reverses DM symptoms has implicated leucine as playing an important role [25].

Arginine, proline and oxoproline, which all decreased in concentration in the diabetic rats, are metabolically closely connected and are downstream products of the urea cycle. Creatinine is also present at lower concentrations in this study. Alterations to urea cycle intermediates in humans [27] and animals [28,13] and urea cycle enzymes in STZ-induced diabetic rats have been reported previously [29]. These changes most likely reflect diabetes-mediated hepatic dysfunction, although altered creatinine metabolism in tissues such as the heart have been reported [28]. Proline has previously been shown in animal models of DM to attenuate the SLC6A20 kidney transporter [30]. Arginine supplementation has been shown to increase brown fat mass and reduce white adipose tissue in Zucker diabetic fatty rats (T2D) and diet-induced obese rats through the enhancement of the proliferation, differentiation, and function of brown adipocytes [31]. In addition, both skeletal muscle mass and whole body insulin sensitivity were enhanced in response to arginine supplementation via mechanisms involving increases in muscle mTOR and nitric oxide signaling [32]. Therefore, the decreased concentrations of arginine (as was observed in this study) are potentially detrimental to normal function.

Other amino acid-related changes included an elevation of 5-methoxytryptophan and a decrease in tryptophan concentrations in diabetic animals; these changes have been found previously in the retina [33]. So the increase in 5-methoxytryptophan we observed in serum could have potential as a biomarker for retinal damage in diabetic retinopathy, though further validation studies are required to assess this potential application. Diabetic animals in this study showed electroretinographic changes associated with diabetes and increased retinal vascular permeability.

Creatine is decreased in this study and this has been observed previously in animal models of DM, but in urine and cardiac tissue. The decrease in cardiac tissue may be related to increased demands of creatine/phosphocreatine for energy production through rapid re-synthesis of ATP from ADP in muscle and tissue with high energy demands (for example, cardiac tissue).

Bile acids have an important role to play in regulation of lipid, glucose and energy metabolism through the farnesoid × receptor and the facilitation of postprandial nutrient absorption by the gut [34]. Our study shows similarities to previous studies highlighting an increased bile acid pool in STZ-induced diabetic rats and other animal models [35]. The sequestering of bile acids can reduce plasma glucose concentrations in diabetic mice [36] so the potential use of bile acid-sequestering drugs in DM is being investigated [37]. Changes in bile acids may also impact on gut microflora-derived metabolites in this study (for example, indolepyruvate and methylindolepyruvate), as has been observed by Connor and colleagues [13].

Changes in other lipid species were also observed. Multiple fatty acids were observed to change, with seven long-chain fatty acids present at higher concentrations in diabetic rats. These changes indicate either an impairment of adipose tissue storage of circulating fatty acids and inhibition of hepatic fatty acid esterification or an alteration to the uptake and utilization of fatty acids via fatty acid beta-oxidation in mitochondria. Both arise from insulin insensitivity causing increased concentrations of non-esterified fatty acids in plasma and ultimately increased beta-oxidation of fatty acids in liver and skeletal muscle mitochondria producing short chain fatty acids and ketone bodies. Increased ketone bodies were observed in this study consistent with insulin deficiency in diabetic animals. Free fatty acids have been shown to cause insulin resistance in many tissues, including cardiac and skeletal muscle [38].

Glycerophospholipids showed both increases and decreases in their concentrations in diabetic rats in this study, implicating changes in cellular membranes and lipoproteins in blood. Here, many lysoglycerophosphocholines (lysoPCs) were decreased in diabetic rats, which indicate a perturbation in the equilibrium between lysoPC production (for example, via phospholipase A2 activity) and lysoPC acetylation (for example, by acetyltransferase enzymes). Although increases in lysoPC concentrations, which are pro-inflammatory mediators, accompany inflammatory responses in DM (for example, with islet autoimmunity in T1D [3]), here we found that serum levels of these pro-inflammatory mediators were decreased. Similar decreases have been observed previously and were related to a shift from lysoPC degradation to glycerophosphocholine production [39].

Sphingolipids were also observed to change and may be related to signaling and plasma membrane changes. Dysfunctional sphingolipid metabolism has been suggested to contribute to metabolic stress in DM and to the pathogenesis of diabetic retinopathy [40,41]. In many of the lipid changes observed there is no direct link between carbon number or degree of saturation and whether these were increased or decreased in STZ-induced diabetic animals.

### Changes in the serum metabolome related to molecular mechanisms of the response to TETA treatment

The second objective of this research was to identify metabolites, classes of metabolites and/or metabolic pathways that are perturbed in DM and return to a pre-diabetes state following treatment with TETA. Some of the already known mechanisms of action and effects of TETA (for a review, see [42]) include: (i) increased urinary copper excretion, (ii) decreased intestinal copper absorption, (iii) telomerase inhibition, (iv) suppression of angiogenic mediators (that is, vascular endothelial growth factor-1, fibroblast growth factor-1, IL-1, IL-6, IL-8 and NFκB), (v) activation of the p38 mitogen-activated protein kinase pathway, (vi) reduced over-expression of Cu/Zn superoxide dismutase, (vii) reversed activation of transforming growth factor-beta and fibrosis in diabetes-induced nephropathy, and (viii) suppressed carbonyl stress in lenses of diabetic rats. However, TETA is likely to have additional mechanisms of action and the objective was to identify other TETA-related changes in the diabetic rats by applying metabolomic technologies.

Multivariate PCA analysis showed no clear indication of metabolic differences between STZ-induced diabetic/TETA-treated and STZ-induced diabetic/untreated rats in study 1 or study 2. Univariate analysis showed one metabolic feature whose relative concentration change was shown to be statistically significant in both study 1 and study 2, putatively identified as hydroxybutanoate and/or methyl-hydroxybutanoic acid. This change most likely relates to the formation of ketone bodies, which is well known in DM.

To further investigate any potential changes in the serum metabolic profile of STZ-induced diabetic rats in the presence or absence of treatment with TETA, pairwise correlation analysis was performed. Correlation analysis was chosen to investigate the complex interaction and regulatory mechanisms of biochemicals (metabolites, proteins, mRNA and genes) in mammalian systems. Metabolites are directly or indirectly correlated to other metabolites in metabolic networks and other direct or indirect correlations between metabolites and other biochemicals are central to the regulation of biological systems. Differences between pairwise correlations were detected without statistically significant changes in the concentrations of correlated metabolites. Therefore, the investigation of these complex correlation networks can provide further inferences about the effect of TETA treatment.

Study 2 was chosen as the sample sizes for all four groups were higher than for study 1 where two groups had only six rats at the end of the study because of death of rats during the study. There were 6,514 245 and 1,965 153 pairwise comparisons in positive and negative ion modes relating to 3,610 and 1,983 metabolic features, respectively. Metabolic features were filtered to provide only those features that show: (a) a high positive or negative pairwise correlation for non-diabetic/untreated rats; (b) a loss of this correlation or a switch from a high positive correlation to/from a high negative correlation for STZ-induced diabetic/untreated rats (that is, a correlation coefficient change >0.5); and (c) a return to a high positive or negative correlation in the STZ-induced diabetic/TETA-treated rats. We found that 30,784 and 4,040 metabolic features showed this pattern in positive and negative ion modes, respectively. To filter the data further, only metabolic features exhibiting this correlation pattern with ten or more different metabolites were investigated further. This provided 857 and 77 metabolic features in positive and negative ion modes, respectively, and related to 506 uniquely identified metabolites. These metabolites are listed in Additional file 1. Of specific interest are multiply charged species, fatty acids and related metabolites, glycerophospholipids, sphingolipids, vitamin D metabolites, sterol metabolites and bile acids.

Specific classes of metabolites were observed as potentially important in defining metabolic changes related to TETA treatment of STZ-induced diabetic rats. Of the unique metabolic features, 337 of 506 (66.7%) were definitively assigned as multiple charged species (predominantly doubly and triply charged species) by the isotopic pattern observed for these metabolic features. Of these 337, 336 species were detected in positive ion mode. These species could be peptides, doubly charged metabolites of TETA (as TETA is a polyamine) or charged non-covalent associations composed of metabolite and Cu (II) with a charge state of +2 (defined as adduct ions). The charge state of some metabolites detected in our study may be influenced by the presence of metal ions (for example, Cu^+ ^and Cu^2+^). Further investigations of these metabolic features showed that the mass difference between isotopic peaks does not relate to expected mass differences that would be observed for charged non-covalent associations composed of metabolite and Cu (II) with a charge state of +2. The mass differences observed did relate to expected mass isotopic differences between ^12^C and ^13^C for doubly and triply charged organic species. These are most probably peptides in view of the hundreds of different features detected (it would not be expected that there would be hundreds of TETA metabolites present at high concentrations), though the absence of TETA metabolites cannot be proven without further targeted studies. It should be noted that 28% of all detected metabolic features in positive ion mode were allocated a multiply charged assignment that is significantly greater than observed in previous animal- or human-based studies in Manchester. This is observed across all four groups and therefore is not a direct result of disease or TETA treatment. However, greater than 20 multiply-charged species were statistically significant between non-diabetic/untreated and STZ-induced diabetic/untreated rats. These results highlight that the methods applied in this discovery study have the potential to identify non-metabolic changes and strengthen the applicability of the method. The changes in the correlation network of peptides are most likely related to changes in proteolysis (or protein catabolism) activity in the diabetic state and then following TETA treatment. Increased proteolysis in insulin resistance and DM has been reported, most likely from the removal of the anti-catabolic effect of insulin [43,44]. There is increased oxidative stress in diabetes and this leads to tissue degeneration and proteolysis. For example, oxidative stress in the retina results in the activation of caspase-3 and apoptosis of endothelial cells and pericytes [45], and inhibition of caspase-1/IL-1beta signaling prevents degeneration of retinal capillaries in DM [46]. Copper chelation will lead to reduced oxidative stress and could prevent caspase activation.

Reversible changes were observed in a number of lipid classes and links between elevated cellular copper levels and lipid metabolism have been reported [47]. These include down-regulation of cholesterol and steroid biosynthesis pathways and fatty acid metabolism. In this study, changes were observed in fatty acid metabolism and sterol metabolism leading to cholesterol and bile acid biosynthesis. Nineteen fatty acids and related metabolites showed changes in this study and included prostaglandins, hydroxyl fatty acids and five fatty acid amines or amides. These TETA-induced changes may be due to decreased oxidative stress. Differences in fatty acid amides may be linked to fatty acid amide hydrolase (FAAH) as polymorphisms of FAAH genes have been linked to obesity and insulin resistance [48] and endocannabinoid system-related genes (of which FAAH is one) have been shown to be effected by insulin dysregulation in adipose tissue [49]. Thirty-seven glycerophospholipids showed changes in this study. A wide range of different glycerophospholipids showed changes, including glycerophosphocholines, glycerophosphoethanolamines, glycerophosphoglycerols, glycerophosphoinositols, glycerophosphoserines, phosphatidate and glycerophosphocholine. These findings indicate a wide-ranging change in glycerophospholipid metabolism related to either cellular membranes or lipoproteins. It has been shown that copper-induced oxidation of lipoproteins affects structure [50] and fatty acid composition [51]. The equilibrium between phospholipase activity and reacylation activity in DM was discussed in the previous section and the changes in several lysoPCs indicate a reversal to higher lysoPC content following treatment. Six sphingolipids show changes, including two sphingomyelins and sphingosine-1-phosphate (S1P). Sphingolipids are important bioactive molecules in signaling pathways involved in apoptosis, proliferation, survival and angiogenesis. These can also regulate oxidant activity. S1P plays an important role in signaling in many disease, including DM [52], and has been shown to regulate beta cell apoptosis [53]. Changes in S1P have been associated with diabetic complications [54], including in kidney nephropathy [55] and the role of oxidative stress in diabetic skeletal muscle [56], and has been shown as a potential biomarker of T1D [40]. These results show that even though clear changes in the concentration of individual metabolites or peptides are not observed in this study, the interactions between metabolites or other biochemical species can be studied (and changes observed) when studying correlation networks of metabolites. This type of analysis provides evidence of metabolism-related changes and changes in proteolysis as a result of TETA-treatment.

## Conclusions

STZ-induced diabetic rats have shown several metabolic changes that have been previously observed and some novel changes that require further investigation. The STZ-induced diabetic rat is an appropriate model system to investigate metabolic changes associated with DM and observed in other animal models of DM, as well as humans. The application of two independent biological studies has highlighted the prevalence of false discovery in these types of studies; many statistically significant changes were observed in only one of two studies, suggesting that they may be false positives. Finally, the metabolic changes associated with TETA treatment have shown that a range of biological mechanisms are implicated and potentially resolved after TETA treatment in diabetic rats, in particular including findings consistent with suppression of proteolysis.

## Materials and methods

All chemicals and reagents applied were of analytical reagent grade or higher.

### Animals and plasma collection

All animal experiments were conducted in accordance with the UK Home Office regulations for the care and use of laboratory animals, the UK Animals (Scientific Procedures) Act (1986), and the ARVO Statement for the Use of Animals in Ophthalmic and Vision Research. Wistar adult male rats were included in the study and were fed with standard laboratory chow and kept in a 12:12 h light:dark cycle. Two independent replicate studies were performed to reduce the probability of reporting false positive observations. The replicate animal and metabolomic studies were separated in time. Animal study 1 was performed from July to October 2009 and study 2 from February to April 2010. For each study, the STZ-induced diabetic group (n = 20) consisted of age-matched animals that received an intraperitoneal injection of STZ (55 mg/kg; Sigma Aldrich, Gillingham, UK) and showed blood glucose levels of ≥30 mmol/L on two consecutive measurements 3 and 6 days after the injection. Assessment of the glycemic state of the animals was carried out by measuring blood glucose concentrations. This method can be easily applied by collecting a small amount of venous blood and is known to correlate well with serum levels of fructosamine and glycosylated haemoglobin. Given that only a small blood sample is required, it alleviates the stress associated with the serial blood sampling required for a glucose tolerance test. Non-diabetic animals (n = 20) were age matched and received an intraperitoneal injection of Na citrate buffer. Ten animals from each group (STZ-induced diabetic and non-diabetic) were treated with oral TETA (20 mg/kg/day; Sigma Aldrich) by gavage from the day after STZ injection until the day before they were sacrificed. Animals were housed in collective cages (maximum of four per cage) and had free access to water and food. Twelve weeks after STZ administration, blood samples were collected from the tail vein in non-fasting animals into 2 ml tubes (Greiner Bio-One Ltd, Stonehouse, UK), placed on ice and subsequently centrifuged at 2,400*g *at 4°C. Serum was separated into 200 μl sub-aliquots and stored at -80°C until analysis. All blood samples were taken between 8:00 and 8:30 am for each individual animal included in each study. The time between blood collection and storage was less than 1 hour for all samples.

### Metabolomics

#### Sample preparation

Samples were randomized before sample preparation. Serum samples were thawed on ice, deproteinized and the sample extract lyophilized in a similar approach as previously described [57]. This process involved addition of 240 μl of methanol to 80 μl of serum in a 2 ml Eppendorf tube followed by vortex mixing (15 seconds) and centrifugation (15 minutes, 13,685*g*). The supernatant was transferred to a separate 2 ml Eppendorf tube and was dried (HETO VR MAXI vacuum centrifuge attached to a Thermo Svart RVT 4104 refrigerated vapor trap; Thermo Life Sciences, Basingstoke, UK). Samples were stored at 4°C until analysis. A pooled QC sample was prepared by the pooling of 30 μl aliquots from each sample and vortex mixing (60 seconds). 80 μl aliquots of the pooled QC sample were deproteinized and lyophilized as described above. Samples collected in animal study 1 and animal study 2 were prepared and analyzed in November 2009 and May 2010, respectively.

#### UPLC-MS analysis

Rat serum extracts and QC samples were analyzed applying an Acquity UPLC system (Waters, Elstree, UK) coupled to an electrospray hybrid LTQ-Orbitrap XL mass spectrometer (ThermoFisher Scientific, Bremen, Germany). All samples were analyzed separately in positive and negative ion modes. The UPLC and MS methods applied have been described previously [57]. QC samples were analyzed for the first ten injections and then every fifth injection. The final two injections were also a QC sample.

#### Data processing and data analysis

Raw data files (.RAW) were converted to the NetCDF format using the File converter program in XCalibur (ThermoFisher Scientific). Deconvolution of the NetCDF format files were performed using the XCMS software, an open-source deconvolution program available for LC-MS data as described previously [58] and each detected metabolic feature was normalized to the QC sample using quality control-robust loess signal correction (QC-RLSC) [57]. Quality assurance (QA) was subsequently performed and only metabolic features that were detected in greater than 60% of all QC samples (from injection 8) and with a relative standard deviation for measured peak areas of <20% were retained for data analysis [57]. All other metabolic features were removed from the dataset and ignored in subsequent data analysis.

All univariate and multivariate analyses were carried out using the Matlab^® ^scripting language [59] and exploratory multivariate analysis was performed using PCA. PCA was performed on data normalized to zero mean and unit variance and the first three PCs were investigated visually.

Univariate analysis was performed using the Mann-Whitney U test, a non-parametric method for assessing whether two independent samples of observations come from the same distribution. No assumption is made of a normal distribution and the test is identical to a one-way analysis of variance (ANOVA) with the data replaced by their ranks. For multivariate analysis all missing values were annotated as 0 and in univariate analysis annotated as 'NaN'. All analyses were performed on data from both ion modes separately but the results have been combined post-data analysis to allow biological interpretation.

#### Pairwise correlation analysis

Pairwise metabolite correlations were calculated for data acquired in study 2 separately for the four different groups (STZ-induced diabetic/TETA-treated, STZ-induced diabetic/untreated, non-diabetic/TETA-treated and non-diabetic/untreated) using the non-parametric Spearman rank correlation method and by applying the bootstrapping method (n = 100). Final results only included comparisons where there were a minimum of 6 versus 6 pair-wise peak correlations. All missing values were annotated 'NaN'. Changes in the correlation network were then calculated by comparison of data from (a) non-diabetic/untreated, (b) STZ-induced diabetic/untreated and (c) STZ-induced diabetic/TETA-treated groups. Metabolic features with a high correlation (greater than +0.5 or less than -0.5) in non-diabetic/untreated and STZ-induced diabetic/TETA-treated rats but with a loss of the correlation coefficient (correlation coefficient change >0.5) in STZ-induced diabetic/untreated rats were retained as biologically important in relation to TETA-treatment of STZ-induced DM. Only metabolic features exhibiting this correlation pattern with ten or more different metabolites were investigated further.

#### Metabolite identification

Metabolic features, characterized by a unique accurate mass and retention time, were putatively annotated according to level 2 the Metabolomics Standards Initiative guidelines [60] applying the PUTMEDID-LCMS identification workflow operating in Taverna [61]. For putatively annotated metabolic features, the accurate mass for each peak was assigned a single or multiple molecular formula matching in mass to the experimentally determined mass with a mass error less than ±5 ppm. Features were subsequently matched to specific metabolites by matching of the molecular formula to metabolites present in the Manchester Metabolomics Database [62]. Further filtering of data, based on expected retention time ranges, was performed. It has been shown that a single metabolite can be detected as multiple metabolic features, each with the same retention time but different accurate mass [62]. Therefore, data analysis can define multiple features of a single metabolite as statistically significant. Also, isomers are detected with the same accurate mass and retention time, and therefore cannot be differentiated and all isomers are reported.

## Abbreviations

DM: diabetes mellitus; FAAH: fatty acid amide hydrolase; IL: interleukin; lysoPC: lysoglycerophosphocholine; mTOR: mammalian target of rapamycin; NF: nuclear factor; PCA: principal components analysis; QC: quality control; S1P: sphingosine-1-phosphate; STZ: streptozotocin; T1D: type 1 diabetes; T2D: type 2 diabetes; TETA: triethylenetetramine; UPLC-MS: ultra performance liquid chromatography-mass spectrometry.

## Competing interests

GJC declares a potential conflict of interest, a non-financial relationship with PhiERA Limited, Auckland, New Zealand. All other authors have no competing interests.

## Authors' contributions

MU and GC jointly conceived the study, jointly performed the experimental design, performed the animal study, jointly analyzed and interpreted the data and jointly prepared the manuscript. MB jointly performed the experimental design and jointly analyzed and interpreted the data. KAH jointly analyzed and interpreted the data. PB jointly conceived the study, jointly performed the experimental design, jointly analyzed and interpreted the data and jointly prepared the manuscript. WBD jointly performed the experimental design, performed the metabolomic study, analyzed and interpreted the data and prepared the manuscript. All authors have read and approved the manuscript for publication.

## Supplementary Material

Additional file 1 **File describing all uniquely identified metabolites showing changes in pairwise correlation coefficients between non-diabetic/untreated and STZ-induced diabetic/untreated rats and changes in pairwise correlation coefficients between STZ-induced diabetic/TETA-treated and STZ-induced diabetic/untreated rats**. Click here for file
